# Fragmentación, heterogeneidad y desafíos del sistema de salud argentino en el contexto de reformas ultraliberales emergentes

**DOI:** 10.1590/0102-311XES012725

**Published:** 2026-08-03

**Authors:** Evangelina Martich, Adelyne Maria Mendes Pereira, Eduarda Ângela Pessoa Cesse

**Affiliations:** 1 Departamento de Ciencias Sociales, Universidad Carlos III de Madrid, Madrid, España.; 2 Escola Nacional de Saúde Pública Sergio Arouca, Fundação Oswaldo Cruz, Fiocruz, Rio de Janeiro, Brasil.; 3 Instituto Aggeu Magalhães, Fundação Oswaldo Cruz, Fiocruz,Recife, Brasil.

**Keywords:** Sistemas de Salud, Desigualdad en Salud, América Latina, Health Systems, Health Status Disparities, Latin America, Sistemas de Saúde, Desigualdades em Saúde, América Latina

## Abstract

El presente trabajo es un estudio de caso que analizó las características de la fragmentación en el sistema de salud argentino y sus implicaciones para las desigualdades en los resultados de salud. El estudio abarca desde 1994, año en que se realizó la última reforma constitucional y se impulsó un proceso de descentralización y desregulación de la salud que profundizó la fragmentación, hasta 2024, marcado por un gobierno que propone reorganizar el sistema de salud implementando medidas de corte ultraliberal que podrían acentuar aún más los problemas de acceso y equidad del sistema. La investigación se realizó a partir de dos dimensiones: (1) contexto político y económico en Argentina; y (2) características de la fragmentación del sistema de salud argentino. Se revisaron y analizaron fuentes secundarias incluyendo artículos científicos, documentos y base de datos oficiales y literatura gris. Los resultados indican que la trayectoria de la fragmentación del sistema de salud argentino se caracteriza por tres fases. La primera fase (1994-2002) fue marcada por la fragmentación vía desregulación y descentralización de funciones en el sistema de salud, con pérdida de la capacidad de coordinación nacional. La segunda (2002-2015) muestra los intentos de disminuir la fragmentación y las desigualdades en la provisión a través de la regulación y programas dirigidos a la población más vulnerable. Fue un periodo importante, aunque la ausencia de cambios estructurales haya colaborado para que la tercera fase (2015-2024) se caracterice por la profundización de la fragmentación, con reducción del rol del Estado y reformas ultraliberales a partir del 2023. El análisis evidencia que el sistema de salud argentino necesita una reorganización que priorice la universalización y acceso equitativo.

## Abstract (en)

This study analyzed the characteristics of fragmentation in the Argentine health system and its implications for inequalities in health outcomes. The study covers the period from 1994, the year in which the last constitutional reform was carried out and a process of decentralization and deregulation of health care was promoted, to 2024, marked by a government that proposes to reorganize the health system implementing ultra-liberal measures that could further exacerbate problems of access and equity in the system. The research was conducted based on the following dimensions: (1) political and economic context in Argentina; and (2) characteristics of the fragmentation of the Argentine health system. Secondary sources were analyzed, including scientific articles, official documents and databases, and gray literature. The results indicate that the trajectory of fragmentation of the Argentine health system is characterized by three phases. The first phase (1994-2002) was marked by fragmentation through deregulation and decentralization of functions in the health system, with a loss of national coordination capacity. The second phase (2002-2015) shows the intention to reduce fragmentation and inequalities in health provision through regulation and programs aimed at the most vulnerable population. It was an important period, but the lack of structural changes contributed to the third phase (2015-2024) being characterized by deepening fragmentation, the reduction of the role of the State and ultra-liberal reforms from 2023 onwards. The analysis shows that the Argentine health system needs to be reorganized to prioritize universalization and equitable access.

## Resumo

O presente trabalho analisou as características da fragmentação no sistema de saúde argentino e suas implicações para as desigualdades nos resultados de saúde. O estudo abarca desde 1994, ano em que se realizou a última reforma constitucional e se impulsionou um processo de descentralização e desregulamentação da saúde que aprofundou a fragmentação, até 2024, marcado por um governo que propõe reorganizar o sistema de saúde implementando medidas de corte ultraliberal que poderiam acentuar ainda mais os problemas de acesso e equidade do sistema. A investigação foi realizada a partir das seguintes dimensões: (1) contexto político e econômico na Argentina; e (2) características da fragmentação do sistema de saúde argentino. Foram analisadas fontes secundárias, incluindo artigos científicos, documentos e base de dados oficiais e literatura cinza. Os resultados indicam que a trajetória da fragmentação do sistema de saúde argentino é caracterizada por três fases. A primeira fase (1994-2002) foi marcada pela fragmentação via desregulamentação e descentralização de funções no sistema de saúde, com perda da capacidade de coordenação nacional. A segunda (2002-2015) mostra as intenções de diminuir a fragmentação e as desigualdades na oferta em saúde por meio da regulamentação e de programas direcionados à população mais vulnerabilizada. Foi um período importante, mas a ausência de mudanças estruturais colaborou para que a terceira fase (2015-2024) se caracterice pelo aprofundamento da fragmentação, pela redução do papel do Estado e pelas reformas ultraliberais a partir de 2023. A análise evidencia que o sistema de saúde argentino precisa de uma reorganização que priorize a universalização e o acesso equitativo.

## Introducción

Los sistemas de salud en Latinoamérica enfrentan importantes retos, asociados, por un lado, a las desigualdades sociales y económicas que marcan la región, y por otro, a las reformas impulsadas desde la década de 1990 orientadas hacia el mercado. Estas reformas ampliaron el sector privado y aumentaron la fragmentación de los sistemas ^1^ ^,^ ^2^.

Si bien hubo cambios importantes en las políticas de salud con la emergencia de gobiernos de coaliciones de izquierdas entre 2000 y 2010 en muchos países de Latinoamérica, con avances en la atención primaria de salud (APS) y reformas hacia la universalidad y la equidad ^3^ ^,^ ^4^, la fragmentación de los sistemas de salud sigue siendo un desafío. Esa fragmentación se profundizó más recientemente, impactando en barreras de acceso relacionadas con la segmentación de la población según afiliación/régimen, a las variaciones de cobertura según capacidad de pago y a las características de la provisión y del financiamiento público y privado ^5^ ^,^ ^6^ ^,^ ^7^.

En Argentina, la fragmentación y segmentación del acceso a la salud han sido rasgos inherentes de su propia configuración institucional, compuesta por tres subsistemas que coexisten: público (que, según datos del último censo nacional del año 2022, cubría alrededor del 36% de la población), seguridad social (46%) y privado (16%) ^8^. El subsistema público de salud abarca a todos los prestadores de salud (a nivel nacional, provincial y municipal), en todos los niveles de atención (atención primaria, secundaria y alta complejidad), ofreciendo acceso libre y gratuito ^9^ ^,^ ^10^.

La seguridad social brinda servicios de salud a los trabajadores del sector formal (activos y jubilados) y a su núcleo familiar directo. La gestión está a cargo de las llamadas Obras Sociales que funcionan como seguros de salud y no constituyen un grupo homogéneo entre sí ^11^ ^,^ ^12^. Este subsector lo integran 291 entidades, compuesto por obras sociales nacionales y provinciales, y el Instituto Nacional de Servicios Sociales para Jubilados y Pensionados (INSSJP) − más conocido como PAMI (Programa de Asistencia Médica Integral), que cubre al 91% de la población con 65 años o más. Las diferencias en la calidad de la cobertura, los recursos disponibles y la modalidad de contratación son muy marcadas entre todos los agentes que componen el subsector de la seguridad social. Cada uno de los cuatro tipos es regulado y controlado por organismos y normativas diferentes, lo que incrementa la fragmentación del sistema de salud incluso en el interior del subsector ^13^.

El subsistema privado comprende a profesionales independientes, prestadores de servicios y las prepagas, que brindan cobertura generalmente a población de ingresos medios-altos y altos, y a su núcleo familiar, siendo el acceso dependiente de la capacidad de pago individual. Se desarrolló en Argentina desde la década de 1960, alcanzando su auge en la década del 1990. En 1994, con la desregulación de las obras sociales, fue permitido que la población escogiera a cuál obra social destinar sus contribuciones, incluso por fuera de su rama de actividad económica y/u optar por transferir sus contribuciones a un seguro privado. En este último caso, a veces las personas acaban además realizando un pago extra (más allá de la contribución obligatoria) de su propio bolsillo para cubrir el valor de la cuota mensual ^14^. 

El objetivo de este artículo es analizar las características de la fragmentación en el sistema de salud argentino y sus implicaciones para las desigualdades en los resultados de salud. Para eso se consideró el período que abarca desde 1994, año en que se realizó la última reforma constitucional y se impulsó un proceso de descentralización y desregulación de la salud que profundizó la fragmentación, hasta 2024, con la llegada del nuevo gobierno de Javier Milei. Este último ha propuesto medidas de corte ultraliberal que podrían acentuar aún más los problemas de acceso y equidad del sistema.

La literatura sobre sistemas de salud ^15^ ^,^ ^16^ presenta evidencias de que reformas que han propuesto medidas como privatizaciones, descentralización y desregulación tienden a aumentar aún más la fragmentación y las desigualdades en el acceso para la población. En este sentido, el estudio del caso argentino se justifica por dos razones. Primero, porque se trata de un país con un sistema de salud caracterizado por una marcada fragmentación que se traduce en resultados de salud desiguales para la población, donde, si bien diferentes gobiernos intentaron mitigar esas disparidades, no han logrado introducir cambios estructurales que reviertan la fragmentación. En segundo lugar, la llegada de un nuevo gobierno al poder ejecutivo nacional, en diciembre del 2023, abrió el debate para una posible reorganización del sistema bajo una óptica ultraliberal. La reorganización en cuestión, además de no reconocer a la salud como un derecho, apela a la priorización de la eficiencia en términos económicos por sobre el fortalecimiento en la prestación de servicios e insumos para reducir las históricas inequidades entre subsistemas y regiones del país. 

Se considera Argentina un caso crítico de marcada fragmentación histórica, con posibles lecciones para otros países de la región. Se espera que el estudio del caso argentino contribuya para la comprensión de aspectos coyunturales e institucionales que condicionan la universalización y la equidad en salud.

## Metodología

Se realizó un estudio de caso en el marco de perspectivas teóricas sobre políticas públicas y sistemas de salud comparados ^17^ ^,^ ^18^. Con foco en la trayectoria de la fragmentación del sistema de salud en Argentina, la investigación se dio a partir de dos dimensiones: (1) contexto político y económico en Argentina; y (2) características de la fragmentación del sistema de salud argentino, como muestra el Cuadro 1. El estudio abarca desde 1994, con la reforma de la constitución que promovió la descentralización sanitaria, y 2024, incluyendo las principales medidas impulsadas por el nuevo gobierno, y el análisis permitió subdividir este período en tres etapas diferentes: 1994-2002; 2002-2015; 2015-2024.

**Cuadro 1:** Matriz de análisis de la fragmentación del sistema de salud y sus implicaciones para las desigualdades en salud.

DIMENSIONES DE ANÁLISIS	COMPONENTES CLAVE DE ANÁLISIS
Contexto político y económico en Argentina	Contexto político: orientación política de los gobiernos y de sus reformas
	Contexto económico: crisis económicas y sus repercusiones, prioridades macroeconómicas
Características de la fragmentación del sistema de salud argentino	Configuración institucional basada en la segmentación de la población según afiliación/régimen
	Variaciones de cobertura y provisión según capacidad de pago
	Medidas hacia la desregulación y de debilitamiento del rol regulador del Estado en la salud
	Variaciones en el gasto en salud (gasto total, público, de la seguridad social y privado, incluyendo el gasto directo del bolsillo)
	Expresiones en las desigualdades en los resultados en salud de la población

Fuente: elaboración propia.

Considerando la influencia de los contextos políticos y económicos en la organización de los sistemas de salud, la primera dimensión incluye la orientación política de los gobiernos y de sus reformas, además de las medidas macroeconómicas implementadas y sus efectos. La segunda dimensión trata de cinco aspectos-clave para la comprensión de la fragmentación del sistema de salud a lo largo del tiempo, abarcando las características de la segmentación de la población según afiliación/régimen; variaciones de cobertura y provisión según capacidad de pago; medidas hacia la desregulación y de debilitamiento del rol regulador del Estado en la salud; variaciones en el gasto en salud (gasto total, público, de la seguridad social y privado, incluyendo el gasto directo del bolsillo); y las desigualdades en los resultados en salud de la población.

El trabajo se basó en una extensa revisión de literatura, donde se recopilaron y analizaron fuentes secundarias de información, artículos académicos, informes oficiales, documentos de organismos internacionales (Organización Mundial de la Salud y Organización Panamericana de la Salud) y publicaciones estadísticas recientes del Ministerio de Salud y del Instituto Nacional de Estadística y Censos (INDEC) de la República Argentina. La búsqueda se llevó a cabo en bases de datos como PubMed, Scopus y Google Scholar, priorizando publicaciones de los últimos diez años. Los criterios de selección incluyeron relevancia temática, rigor metodológico y relación directa con el sistema de salud argentino. 

El análisis de la información se abordó desde un enfoque cualitativo, buscando identificar patrones, contrastes y relaciones clave en los datos obtenidos. Se analizaron trabajos previos sobre desigualdades regionales, gestión del sistema de salud y resultados en salud. 

Este trabajo presenta algunas limitaciones metodológicas, como ser el uso exclusivo de fuentes secundarias y la escasez de datos subnacionales, que limita la comparación entre regiones, algo que espera poder profundizar en futuras investigaciones. Del mismo modo, se espera poder incorporar al análisis resultados de algunas de las medidas implementadas durante el gobierno actual.

## Resultados

En la primera fase (1994-2002) se evidenciaron dos características principales de la fragmentación: la desregulación y la descentralización de funciones en el sistema de salud, con pérdida de la capacidad de coordinación nacional. En la segunda fase (2002-2015), se destacaron los intentos de disminuir la fragmentación vía regulación y de reducir las desigualdades en la provisión a través de programas dirigidos a la población más vulnerable. Fue un periodo importante, aunque la ausencia de cambios estructurales en la configuración del sistema haya colaborado para que la tercera fase identificada (2015-2024) esté marcada por la desregulación y profundización de la fragmentación y desigualdades en la provisión de los servicios, con la presencia de reformas ultraliberales a partir del 2023.

Las tres fases antes mencionadas, lejos de ser categorías estáticas, presentan ambivalencias y tensiones. Sería necesario profundizar este trabajo para poder analizar los detalles a los cuales responden esas tensiones que, por ejemplo, han hecho que, aun con intentos de revertir la fragmentación del sistema, no se hayan realizado reformas estructurales que permitieran revertirla.

El Cuadro 2 presenta una síntesis detallada de las principales características del contexto general y de la fragmentación de la salud en Argentina.

**Cuadro 2:** Trayectoria de la fragmentación del sistema de salud y sus implicaciones para las desigualdades en salud en Argentina en el período del 1994 al 2024.

MARCO TEMPORAL	CONTEXTO POLÍTICO Y ECONÓMICO EN ARGENTINA	CARACTERÍSTICAS DE LA FRAGMENTACIÓN DEL SISTEMA DE SALUD ARGENTINO
Fase 1: fragmentación vía desregulación y descentralización de funciones en el sistema de salud argentino		
1994-2002 Carlos Menem (1989-1999) Partido Justicialista (PJ) Fernando De la Rúa (1999-2001) Alianza UCR-Frepaso	- Escenario inicial: hiperinflación, crisis económica, política e institucional - Medidas: hasta 1999, apertura económica, privatización de empresas estatales, liberalización del mercado y convertibilidad de la moneda (fijó el valor del peso en relación con el dólar). Entre 1999 y 2001, ajuste fiscal, recesión económica y desvalorización de la moneda - Efectos: aumento del desempleo, precarización laboral y pérdida del poder adquisitivo de la población (que se profundiza en 1999); salida anticipada del presidente antes de acabar su mandato en diciembre del 2001	- Segmentación de la población según los tres regímenes (público, seguridad social y privado), con aumento de la población atendida por el subsector público (por la crisis económica y del empleo) - Aumento en las variaciones de cobertura y provisión según capacidad de pago. Tentativa de reducir las diferencias en la provisión médica y odontológica entre la seguridad social y las prepagas a través del Programa Médico Obligatorio (PMO, 1996) - Desregulación y descentralización de la salud, justificada bajo la idea de reducir el gasto público y el déficit fiscal (transfiriendo responsabilidades a los otros niveles de gobierno). Desregulación de las obras sociales (1997) - Variaciones en el gasto en salud, con descenso de aportes a la seguridad social (por el aumento del desempleo) - Inicio de un proceso de fusión de empresas que se mantiene hasta la actualidad (fuerte concentración en manos de pocas compañías)
Fase 2: intentos de disminuir la fragmentación vía regulación y de reducir las desigualdades en la provisión a través de programas dirigidos a la población más vulnerable		
2002-2015 Eduardo Duhalde (2002-2003) Partido Justicialista (PJ) Néstor Kirchner (2003-2007) Frente para la Victoria (PJ) Cristina Fernández (2007-2015) Frente para la Victoria (PJ)	- Escenario inicial: gobierno de transición tras la salida prematura del presidente De la Rúa. En 2003, crisis política e institucional y bajos niveles de apoyo inicial Medidas: - En 2002-2003, fin de la convertibilidad y la devaluación del peso; implementación de políticas sociales destinadas a contener la crisis, el elevado desempleo y la pobreza - Entre 2003 y 2007, recuperación económica, crecimiento del empleo, superávit fiscal y renegociación de deuda externa; fortalecimiento del papel del Estado, la inversión pública y el desarrollo industrial - Entre 2007 y 2025: continuidad del modelo económico basado en el consumo interno y la intervención estatal; y nacionalización de varias empresas estratégicas - Efectos: creció el nivel de empleo al inicio del período, disminuyendo hacia el final; aumento de la inflación y dificultades para el acceso al mercado financiero internacional	- Segmentación de la población según los tres regímenes (público, seguridad social y privado) - Ampliación del rol del Estado en la coordinación a través de la revitalización del Consejo Federal de Salud para reducir la fragmentación entre las jurisdicciones federal, provincial y municipal - Regulación de las prepagas a través de la *Ley 26.682*. - En el subsector de la seguridad social no se introdujeron cambios significativos en la regulación o provisión - Ausencia de cambios estructurales para reducir la fragmentación. Entre 2002 y 2003, implementación de programas focalizados en grupos y/o poblaciones vulnerables: Programa Médico Obligatorio de Emergencia (PMOE) basado en la atención primária de salud como estrategia de organización de los servicios de salud y privilegiando acciones de prevención; Programa Remediar para promover acceso a medicamentos básicos a la población sin ningún tipo de cobertura; y Programa Nacer/Sumar para brindar aseguramiento a mujeres embarazadas y sus hijos con cobertura exclusiva en el subsector público - Acerca de la provisión en el subsector público: entre 2003 y 2007, se creó el Programa Nacional de Salud Sexual y Procreación Responsable (2003), el Plan Federal de Salud (2004-2007) que promovió las redes de atención planteando como eje la Atención Primaria, y se fortaleció el Programa Remediar+Redes. Entre 2007 y 2015: se fortalecieron y ampliaron el Programa Nacional de Salud Sexual y Procreación Responsable, Remediar y Plan Nacer/Sumar (a partir de 2012, extendió la cobertura a todas las mujeres adultas que no tengan seguro de salud) - Variaciones en el gasto en salud: se incrementó el presupuesto del sector en 2%, alcanzando el 6.20% del Producto Interno Bruto en 2009
Fase 3: desregulación y profundización de la fragmentación y desigualdades en la provisión de los servicios en el sistema de salud argentino		
2015-2024 Mauricio Macri (2015-2019) Cambiemos (PRO-UCR-CC) Alberto Fernández (2019-2023) Frente de Todos (PJ) Javier Milei (2023-) La Libertad Avanza	- Escenario inicial: cambio de orientación económica al inicio del período; giro ultraliberal al final Medidas: - Entre 2015 y 2019, mayor apertura económica, reducción del déficit fiscal y aumento del endeudamiento externo - Entre 2019 y 2023, gestión condicionada por la pandemia del COVID-19 - A partir del 2023, fuerte reducción del gasto público y eliminación de regulaciones económicas - Efectos: aumento de la inflación, disminución del poder adquisitivo de la población y del empleo formal, y aumento de los niveles de pobreza; a partir de 2023, aumento en los niveles de desempleo, empleo informal, pobreza e indigencia entre la población	- Segmentación de la población según los tres regímenes (público, seguridad social y privado) - Se profundizaron las variaciones de cobertura y provisión según capacidad de pago - Reducción del rol del Estado en la regulación y ampliación de la descentralización de funciones a las provincias. Entre 2015 y 2019, el Ministerio de Salud federal se redujo a la categoría de secretaría con pérdida de autonomía y presupuesto. En 2019, el Ministerio de Salud fue restablecido - Entre 2020 y 2023, la gestión sanitaria estuvo fuertemente enfocada en la pandemia del COVID-19: aumento de camas de UTI, respiradores y campaña de vacunación - A partir de 2023, reformas ultraliberales: debilitamiento del rol regulador del Estado y la descentralización en salud, aumentando la fragmentación y desigualdad entre los subsectores - Variaciones en el gasto en salud, con reducción del presupuesto para políticas y programas nacionales a partir del 2023, dificultando el acceso a los servicios de salud, medicamentos, vacunas y otros insumos

Fuente: elaboración propia con base en Ballesteros ^19^, Torres ^20^, Kessler ^22^, Chiara ^25^, Alonso & Di Costa ^26^, Machado et al. ^34^, Salvia & Tissera ^53^, Ramacciotti ^66^.

### Fase 1 (1994-2002): fragmentación vía desregulación y descentralización de funciones en el sistema de salud argentino

En la década de 1990, muchos países de Latinoamérica implementaron transformaciones institucionales del Estado, redefiniendo su papel a partir de las reformas promovidas por el Consenso de Washington. En el sector de la salud, implicó la profundización de la descentralización, desregulación y privatización de algunos servicios en distintos países de la región. 

En Argentina, el presidente Carlos Menem asumió su mandato en 1989 en un escenario de hiperinflación, crisis económica y necesidad de estabilización de la economía. Su gestión se enfocó en medidas de apertura económica, privatización de empresas estatales, liberalización del mercado y convertibilidad de la moneda (fijó el valor del peso en relación con el dólar). El gobierno consiguió una cierta estabilidad inicial y crecimiento económico, pero a mediano plazo derivó en aumento del desempleo, precarización laboral y pérdida del poder adquisitivo de la población, situación que se profundiza al final del mandato.

En salud, se consolidaron procesos de desregulación y descentralización, justificados bajo la idea de que así se superarían problemas de gestión nacional y el sistema se tornaría más capilar y con mayor capacidad de respuesta a las necesidades de salud de la población. En la práctica, se trató de una estrategia para reducir el gasto público y el déficit fiscal transfiriendo competencias y funciones del nivel nacional a las provincias y municipios. Tal es así que, si se compara la cantidad de establecimientos asistenciales del subsector público entre 1980 y 1995, los de nivel municipal aumentaron en un 28,7% ^19^.

En cuanto a la desregulación de las obras sociales, se les permitió a los beneficiarios de la seguridad social la libertad de elección de obra social. Ello hizo que los niveles de inequidad aumentaran, facilitando la salida de usuarios de niveles de ingresos superiores para el sector privado de planes de salud (prepagas). Esto conllevó la retirada de contribución para el fondo solidario (subsidio automático para cubrir servicios de alto costo) y rompió con la lógica institucional solidaria del sector.

Además, el sector de la seguridad social se vio afectado por el aumento del desempleo, perdiendo afiliados. Entre los años 1993 y 1997, los beneficiarios de obras sociales nacionales pasaron de 17.369.191 a 15.842.245 ^20^. Esos beneficiarios que, como consecuencia de la pérdida de empleos formales, dejaban de tener cobertura en la seguridad social, pasaron a demandar servicios del subsector público.

En 1996 se creó el Programa Médico Obligatorio (PMO) que buscaba establecer un mínimo de prestaciones que involucraran a todos los subsectores de la salud. Sin embargo, la fragmentación normativa provocó desigualdades en la aplicación, lo que, sumado a la crisis económica, aumentó la heterogeneidad de servicios, la desigualdad en el acceso para los usuarios y dificultó la coordinación de políticas sanitarias en el país ^13^.

El subsector privado (prepagas) perdió afiliados por la crisis económica y comenzó un proceso de fusión y concentración de empresas que se mantuvo a lo largo de todo el período de este análisis. Ello llevó a que, en 2022, apenas cinco empresas de medicina prepaga (Swiss Medical, Galeno, Omint, Medicus y Medife) concentraran todo el mercado argentino. Además, la cobertura territorial es marcadamente desigual, con el 70% de las personas cubiertas por prepagas residiendo en la Ciudad Autónoma de Buenos Aires, mientras que en el norte argentino no supera el 11% ^20^. 

Entre los años 1999 y 2001, la gestión del presidente De la Rúa estuvo marcada por una profunda crisis social, recesión económica, inestabilidad política y fragilidad institucional que acabó con su salida anticipada en diciembre del 2001, antes de finalizar su mandato. La crisis generalizada que atravesaba el país impactó en el financiamiento del sector salud. El gasto en salud como porcentaje del PIB (producto interno bruto) cayó un 0,82% entre los años 1995 y 2000 ^21^, mientras que el gasto público sobre el total del gasto en salud también perdió participación. El subsector de la seguridad social atravesaba una crisis de financiamiento sin precedentes, en este caso por el aumento del desempleo, que alcanzó 21,5% en 2002 ^22^, el valor más alto que se tiene registro en la historia del país. El subsector privado de prepagas se estima que perdió alrededor del 17% de sus afiliados entre los años 2001 y 2003 ^20^. Todo lo anterior provocó que el subsector público se vea sobrecargado: entre 1997 y 2001 creció un 18% la población que solo contaba con cobertura pública de salud ^23^.

### Fase 2 (2002-2015): tentativas de disminuir la fragmentación vía regulación y de reducir las desigualdades en la provisión a través de programas dirigidos a la población más vulnerable

El gobierno de transición del presidente Eduardo Duhalde (2002-2003) se enfocó en la recuperación económica posterior al colapso de diciembre del 2001, el fin de la convertibilidad y la devaluación del peso. El período estuvo marcado por un fuerte aumento del desempleo, la pobreza y la exclusión social, por lo que el gobierno se enfocó en la implementación de políticas sociales destinadas a mitigar los impactos de la crisis y estabilizar el país. Desde el 2002 hasta el 2009, se otorgaron 7 millones de nuevas protecciones sociales, como la extensión de la jubilación, pensiones no contributivas, asistenciales y especiales, y la Asignación Universal por Hijo. En total, el número de prestaciones se duplicó con respecto a las de 1997 ^22^.

A comienzos del año 2002, se incrementó el presupuesto anual en salud mediante recursos fiscales de emergencia y la reorientación de créditos internacionales ya otorgados, creándose varios programas nacionales que significaron una fuerte política redistributiva ^23^. Se estableció el Programa Médico Obligatorio de Emergencia (PMOE) basado en la atención primaria como estrategia de organización de los servicios de salud y privilegiando acciones de prevención. También se creó el Programa Remediar, cuyo objetivo era promover el acceso a los medicamentos para la población sin ningún tipo de cobertura. Se realizaban compras centralizadas y se enviaban botiquines estandarizados de medicamentos genéricos directamente a los centros de atención primaria de todo el país. La logística de organización se basaba en la red de servicios y establecía canales de comunicación entre los tres niveles de gobierno, inaugurando así un modelo de federalismo sanitario con criterios territoriales inexistentes hasta entonces. Además, se sancionó la ley de prescripción de medicamentos por el nombre genérico, la cual incentivó la demanda, pero no promovió la oferta de estos productos, ni estableció precios máximos de salida al mercado ^24^.

Se creó también el Programa Nacer/Sumar para brindar aseguramiento a mujeres embarazadas y sus hijos con cobertura exclusiva en el subsector público. Inicialmente, se implementó en nueve provincias del Norte del país (Chaco, Corrientes, Misiones, Formosa, Santiago del Estero, Tucumán, Salta, Jujuy y Catamarca), que eran las que presentaban los peores indicadores sociosanitarios. El Plan Nacer implementó un modelo de gestión donde el nivel federal financia (transfiriendo recursos a las provincias), coordina y supervisa, mientras que las provincias ejecutan. Si bien este programa le dio una mayor participación al Estado Nacional, no cambiaron las reglas institucionales del funcionamiento del sistema y las características de fragmentación y segmentación permanecieron ^25^.

El subsector de la seguridad social lentamente comenzó a recuperar el número de aportes obligatorios (por aumento de la población con empleo formal) y el aumento del salario real. Desde el 2003, se verificó un incremento del trabajo formal (considerando asalariados registrados e independientes registrados en la seguridad social) y en 2010 el número de asalariados registrados en el sector privado era superior en 43% al mejor momento de los años 1990 ^22^. 

En el año 2003, Néstor Kirchner asumió la presidencia con bajos niveles de apoyo inicial (su contrincante, en la segunda vuelta electoral, renunció antes). Su gestión estuvo marcada por la recuperación económica, crecimiento del empleo, superávit fiscal y renegociación de deuda externa. Además, avanzó en el fortalecimiento del papel del Estado, la inversión pública y el desarrollo industrial ^26^.

En salud, se revitalizó el Consejo Federal de Salud buscando lidiar con un doble frente de fragmentación del sistema: por un lado, en la coordinación de políticas de salud entre jurisdicciones federal, provincial y municipal; por el otro, con la propia fragmentación que caracteriza al sistema en tres subsistemas y específicamente al subsector de la seguridad social que a priori es muy fragmentado en su interior, y al subsector privado que presenta marcadas diferencias entre las diversas regiones del país, como antes mencionado ^27^. Además, se implementó el Plan Federal de Salud (2004-2007), que promovió las redes de atención planteando como eje la atención primaria. Se fortalecieron algunos programas ya existentes, como el Remediar + Redes ampliándolo, y se creó el Programa Nacional de Salud Sexual y Procreación Responsable (2003), promoviendo el acceso a servicios e insumos anticonceptivos. 

Si bien se articularon medidas desde el Estado nacional en salud, no se introdujeron reformas estructurales en el sistema de salud, ni cambios en lo que respecta a los subsectores de la seguridad social ni de las prepagas. Tanto es así que la fragmentación permaneció.

En 2007, asumió la presidencia Cristina Fernández, que gobernó durante dos mandatos consecutivos (2007-2015) y dio continuidad al modelo económico basado en el consumo interno, la intervención estatal y la nacionalización de varias empresas estratégicas. En salud, se incrementó el presupuesto 2%, alcanzando el 6,2% del PIB en 2009 ^26^. Durante su gestión se mantuvieron y fortalecieron algunos programas nacionales, como por ejemplo la extensión en 2012 del Programa Nacer/Sumar, brindando cobertura a todas las mujeres adultas que no tuviesen seguro de salud ^25^.

En este período, se avanzó en la regulación de las prepagas a través de la *Ley 26.682/2011* ^28^. Sin embargo, las prepagas continuaron imponiendo periodos de carencia, rechazando beneficiarios en función de enfermedades preexistentes, por la edad, el sexo u otro tipo de restricciones ^9^. La *Ley 26.682/2011* ^29^ fue reglamentada por el *Decreto 193* ^30^ del mismo año, y más tarde por el *Decreto 66/2019* ^31^ y por el *Decreto 743/2022* ^32^. Ambos introdujeron actualizaciones, entre ellas, la creación del Registro Nacional de Entidades de Medicina Prepaga (RNEMP) y un padrón nacional de usuarios.

El nivel de empleo que había aumentado al inició de la gestión disminuyó hacia el final, lo que, sumado al aumento de la inflación y a las insatisfacciones de la población, condujo a un cambio de rumbo en el país. 

### Fase 3 (2015-2024): desregulación y profundización de la fragmentación y desigualdades en la provisión de los servicios en el sistema de salud argentino

En 2015, asume la presidencia Mauricio Macri con una orientación económica hacia una mayor apertura, reducción del déficit fiscal y aumento del endeudamiento externo. Consecuentemente, hubo un aumento de la inflación, disminución del poder adquisitivo de la población y aumento de los niveles de pobreza. 

En salud, el Ministerio de Salud Federal se redujo a la categoría de secretaría bajo la órbita del Ministerio de Desarrollo Social, perdiendo autonomía y presupuesto. En consecuencia, hubo más fragmentación, con más descentralización de funciones a las provincias y menos capacidad de coordinación nacional. Para el año 2018, el gasto público en salud como porcentaje del PIB alcanzó 2,5%, siendo el valor más bajo de los últimos años. Cuando el gasto institucional cae, el privado tiende a aumentar, de hecho, ese mismo año el gasto privado en salud como porcentaje del PBI alcanzó los 4,2% ^33^.

En diciembre de 2019, Alberto Fernández llega al poder y, al asumir su mandato, restablece el Ministerio de Salud. Su gestión estuvo condicionada por la pandemia del COVID-19 ^34^. 

En el año 2020, el gasto total en salud con relación al PBI alcanzó el 10,9% ^33^. Dentro de ese total, el gasto público aumentó su participación (3,1%) en los años 2020 y 2021 en relación con los años previos. En este período, se aumentó el número de camas UTI, respiradores y, más tarde, se implementó la campaña de vacunación. A pesar de ese incremento del gasto del gobierno nacional con salud, continuó por debajo del gasto de la seguridad social (4% en 2020 y 3,4% en 2021) y del privado (3,8% en 2020 y 3,9% en 2021), como en todo el período. Por otro lado, y como parte de la descentralización de la gestión sanitaria en el país, cabe mencionar que son las provincias las que presentan mayor proporción relativa de gasto en salud, ya que tienen la competencia de brindar servicios de salud a la población.

**Figura 1:**
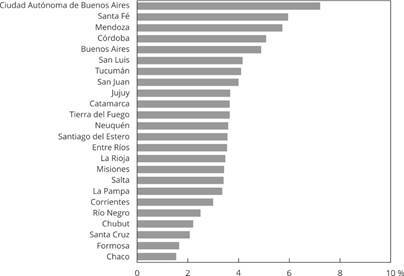
Gasto de bolsillo como porcentaje del ingreso del hogar por provincias.

El gasto de bolsillo como porcentaje del gasto total en salud alcanzó el 24% en el año 2020 y se compone de tratamientos ambulatorios (consultas y copagos); pagos de servicios de internación; medicamentos; insumos y equipamientos (lentes, prótesis, etc.); y cuotas de prepagas ^35^. El gasto de bolsillo presenta diferencias según el tipo de cobertura de la población: el sector privado concentra el mayor porcentaje (10%); está en 4% en el segmento de población cubierto por la seguridad social; y alcanza el nivel más bajo (3%) entre la población con cobertura exclusivamente pública. Además, es posible identificar diferencias territoriales, como se muestra en la Figura 1 (según la última información disponible) ^35^.

Fuente: adaptado de Maceira & Jiménez ^35^.

El mayor gasto de bolsillo se produce en la capital del país, lo que coincide con el distrito donde se encuentra la mayor cantidad de población cubierta por el subsector de la medicina prepaga ^35^.

El gobierno de Alberto Fernández enfrentó altos niveles de inflación (que no logró controlar a lo largo de su mandato), aumento del empleo informal y el desempleo, acabando su gestión con muy bajos niveles de apoyo entre la población.

Javier Milei asumió la presidencia de Argentina en diciembre del 2023, introduciendo un fuerte cambio en la orientación económica y política. Desde entonces, su gestión se ha caracterizado por la reducción del gasto público (eliminando ministerios y programas) y del déficit fiscal, junto con la eliminación de regulaciones económicas. En el primer año de su mandato se produjeron aumentos en los niveles de desempleo (pasó del 5,7% en el 3º trimestre de 2023 al 6,9% en el 3º trimestre de 2024) y pobreza (pasó de 40,1% en el 1º semestre de 2023 al 52,9% en el 1º semestre de 2024) ^36^ ^,^ ^37^.

En salud, el gobierno de Javier Milei proyecta una serie de cambios en el sistema desde una óptica ultraliberal. La concepción ultraliberal de la salud desplaza la responsabilidad de los Estados en garantizar acceso a los servicios e insumos para la población hacia la responsabilidad individual, donde cada uno debe buscar respuestas según su capacidad de pago ^38^ ^,^ ^39^. En nombre de la libertad de elección y de una supuesta eficiencia en la prestación de los servicios, se oculta la mercantilización del derecho a la salud.

Con la aprobación de la Ley Bases en junio de 2024, el actual gobierno argentino avanzó en su plan de desregulación del Estado ^40^ ^,^ ^41^, sin contemplar ninguna medida protectora para el derecho a la salud de la población. A partir de esto podrían producirse una serie de consecuencias institucionales para el sistema sanitario que, directa o indirectamente, impactarían negativamente en los resultados de salud de la población.

Entre los cambios proyectados con impactos sobre las desigualdades, se puede mencionar la profundización de la descentralización, como una forma de desplazar la toma de decisiones a las provincias. La devolución de competencias, sin ir acompañada de una transferencia de recursos, podría aumentar aún más la desigual distribución de la estructura que ya tiene el sistema sanitario argentino, como ya sucedió en la década de los años 1990. La descentralización en salud, como cualquier otro proceso político que involucra distribución del poder en la toma de decisiones, requiere identificar problemas y costos del ámbito subnacional para hacer que la política sanitaria realmente sea permeable a las necesidades de salud de la población ^42^. 

La actual administración nacional argentina planteó la reducción del gasto público como un fin en sí mismo. La descentralización de la salud se encuadra dentro de este objetivo. Muchas localidades alejadas de grandes centros urbanos carecen de infraestructura adecuada e incluso de personal técnico capacitado. Sin inversión federal, la descentralización podría exacerbar aún más esas inequidades regionales del sistema y provocar que los gobiernos provinciales se endeuden ^43^.

También prevé el desfinanciamiento de programas nacionales, con recortes presupuestarios que van del 22% al 55% en áreas claves como HIV, hepatitis, salud sexual y reproductiva y acceso a medicamentos ^44^. Algunos programas, como es el caso del Plan Nacional de Prevención del Embarazo no Intencional en la Adolescencia (Plan ENIA), han sido directamente discontinuados ^45^. Esto conlleva riesgos y consecuencias directas para la salud de la población, ya que excluye aún más a sectores vulnerables y aumenta el ya elevado gasto de bolsillo.

Durante la primera década de los años 2000, se implementaron una serie de programas nacionales (Cuadro 1) que buscaban mitigar los efectos de la gran crisis que atravesó el país en 2001 y a su vez proveer servicios e insumos para la salud para la población independientemente de su tipo de cobertura (pública, seguridad social, privada). Reducir y/o eliminar esos programas es una forma de privatizar el acceso para determinados servicios y, consecuentemente, aumentar la fragmentación y desigualdad. Trabajos anteriores indican que aumentar los niveles de privatización de los sistemas de salud conlleva a que enfermarse pueda convertirse en un factor de empobrecimiento para la población. Además, podría provocar que las personas dejen de buscar servicios médicos cuando los necesitan por incapacidad de pago y se enfrenten a un empeoramiento de su condición de salud, poniendo incluso en riesgo la propia sostenibilidad del sistema ^46^. 

## Discusión

En Latinoamérica, los procesos de fragmentación y segmentación se sitúan en un marco más amplio de reformas neoliberales en los sistemas de salud que contribuyeron para el debilitamiento de la salud como derecho social y el surgimiento de modelos que combinan la idea de universalización con prácticas de provisión selectiva ^47^ ^,^ ^48^ ^,^ ^49^ ^,^ ^50^. La literatura sobre las reformas de los sistemas de salud en Latinoamérica resalta efectos negativos del neoliberalismo, principalmente a través de la reducción del gasto público, la privatización y la mercantilización de los servicios de salud ^47^ ^,^ ^48^. Estas medidas han conducido a una mayor desigualdad en el acceso a la salud, una disminución en la calidad de los servicios públicos y una creciente preocupación por la eficiencia y la rentabilidad por sobre la salud de las personas ^49^ ^,^ ^50^.

En Colombia, por ejemplo, se adoptó una concepción mercantil de la salud, que aumentó la participación del sector privado y la focalización del financiamiento público en los sectores más pobres - lo que acentuó aún más la fragmentación que tenía el sistema ^15^. En Chile, ya desde la década de los años 1980, se profundizó una reforma neoliberal de la salud, promoviendo una intensa articulación entre Estado y mercado, generando una marcada transformación en la estructura y funcionamiento del sistema ^16^.

En Argentina, el escenario es de una fuerte fragmentación y segmentación que se manifiesta en varios aspectos ^10^ ^,^ ^28^ ^,^ ^51^ ^,^ ^52^ ^,^ ^53^ ^,^ ^54^. Por un lado, la propia configuración institucional del sistema de salud, donde coexisten varios subsistemas, con diferentes modalidades de financiamiento y prestación de servicios destinados a diversos segmentos de población ^54^. Por otro lado, la condición laboral de la población hace que las personas accedan a diferentes tipos de cobertura sanitaria según trabajen de manera formal o informal ^28^. 

También es posible identificar fragmentación en un sentido normativo, ya que son diferentes los marcos regulatorios de cada subsistema: el Ministerio de Salud de la Nación ejerce rectoría nacional; la Superintendencia de Seguros de Salud regula las obras sociales nacionales y prepagas; las obras sociales provinciales son reguladas por la provincia a la cual pertenecen; y además existe otro tipo de obras sociales que poseen regímenes especiales ^10^. Todo esto ha producido, a lo largo de décadas, que la cobertura de servicios ofrecida en cada uno de los agentes del sistema sea diferente y cree acceso diferenciado para la población.

Por último, es posible identificar una fragmentación de tipo territorial, que se manifiesta en una desigual distribución de recursos entre las diferentes regiones del país, tanto de camas como de disponibilidad de centros asistenciales y de profesionales, con una fuerte concentración en el centro del país. Por ejemplo, en toda Argentina en 2022 había 175.419 médicos, lo que se traduce en unos 382 profesionales cada 100 mil habitantes. La región central es la que concentra mayor número de profesionales (443 cada 100 mil), mientras que, en el noreste, región más desfavorecida económicamente, hay 49% menos de médicos (apenas 228 cada 100 mil habitantes) ^40^. Estas diferencias regionales repercuten en resultados desiguales en la salud de la población. Según los últimos datos disponibles (2021), en la Ciudad Autónoma de Buenos Aires, la mortalidad infantil fue de 4,6 cada mil nacidos vivos, mientras que en Formosa (provincia de menos recursos económicos) alcanzó los 11,6 ^33^. La mortalidad materna también presenta una enorme diferencia entre regiones del país. En el año 2021, en la Ciudad Autónoma de Buenos Aires, fue de 2,7 cada 10 mil nacidos vivos, mientras que en la provincia de Santiago del Estero se alcanzaron los 19 cada 10 mil nacidos vivos ^33^.

Sobre la prevalencia de enfermedades crónicas, como la diabetes, hay estudios que indican que, además de diferencias regionales, se identifican diferencias dentro de la propia capital del país, aumentando de 11,4% a 15,5% en personas con menores ingresos ^41^. Justamente en el caso de las enfermedades crónicas, su evolución depende, en gran parte, del diagnóstico a tiempo y el tratamiento adecuado ^55^, lo que está directamente relacionado con el acceso que la población tenga a servicios de salud y programas específicos de seguimiento.

El sobrepeso y la obesidad infantil son un importante problema de salud pública que ha ido en aumento. En América Latina y el Caribe, el sobrepeso en niños, niñas y adolescentes es de 30,6%, muy por encima de la prevalencia mundial del 18,2%. Argentina se encuentra entre los tres países con mayor obesidad infantil de la región. Las cuestiones relacionadas con la malnutrición, entre las que se encuentra la obesidad infantil, son más marcadas en las provincias del norte y noreste del país ^56^.

Otro indicador relevante es la prevalencia del embarazo adolescente, que se redujo en un 60% entre 2018 y 2023, y colocó a la Argentina a la vanguardia en la región ^57^. El abordaje consistió en la implementación del Plan ENIA, que, desde un enfoque de derechos, promovía el acceso a métodos anticonceptivos de larga duración (como el implante subdérmico) y el compromiso del personal sanitario a través de la implementación de consejerías para la prevención del embarazo no intencional ^58^. Además, Argentina, junto con Uruguay, son los únicos países de la región donde se ha legalizado el acceso al aborto a través de la ley de interrupción voluntaria del embarazo (*Ley 27.610*/2020) en diciembre del 2020. A pesar de estos avances a nivel nacional, continuaron siendo muy marcadas las diferencias entre provincias del norte y el centro del país ^59^.

Otra cuestión central son los recursos humanos en salud, sus condiciones de trabajo y distribución, tanto entre los diferentes sectores que integran el sistema sanitario como a nivel territorial en la extensa superficie del país. Existe una gran heterogeneidad entre provincias y municipios, zonas urbanas y rurales. El personal sanitario tiene diferentes modalidades de contratación y regímenes de empleo, sufriendo con el pluriempleo y desiguales salarios. Esto aumenta la estratificación en el acceso, que tiene un carácter social y territorial y está directamente relacionado con las condiciones socioeconómicas de las personas. Pero, además, atenta contra una de las lógicas fundamentales de las políticas sociales en general, y de salud en particular, que es justamente disminuir las desigualdades entre la población ^60^.

Con base en lo expuesto, dos lecciones se pueden destacar del caso argentino. La primera se refiere a la función de la fragmentación del sistema de salud como mecanismo de reproducción de desigualdades. En el contexto neoliberal, las poblaciones con menor renta y regiones más vulnerables siguen siendo aquellas con peores resultados en salud y más dificultades de acceso a los servicios, tanto en Argentina como en otros países de la región ^61^ ^,^ ^62^.

Es importante tener en cuenta que la fragmentación del sistema de salud argentino no solo se explica por fallas institucionales, sino que además podría responder a racionalidades del mercado donde la eficacia y la competencia desplazan la lógica del derecho a la salud y desdibujan responsabilidades entre los diferentes subsistemas. A su vez, la existencia de múltiples actores que operan dentro del sistema sin sinergia puede generar ineficiencia en la asignación de los recursos y/o superponer funciones ^63^. Un sistema de salud cuyas partes no trabajan interconectadas dificulta la coordinación, la continuidad de los cuidados y obliga al paciente a transitar por diferentes instancias desconectadas, pudiendo incurrir en interrupciones en su tratamiento, pérdida de información clínica relevante o falta de seguimiento adecuado ^64^. 

En un análisis de seis países de la región, Göttems & Mollo ^47^ discuten las implicaciones de las recomendaciones de organismos internacionales pautadas en el Consenso de Washington sobre las reformas de los sistemas de salud y evidencian que las agendas difundidas tensionan y limitan cambios estructurales. Esto puede explicar parcialmente por qué, incluso en contextos de coaliciones de gobierno progresistas, se mantienen características como la privatización o la fragmentación. Además, existen los intereses del mercado en salud establecidos en cada país, que colaboran para las ambivalencias institucionales en las políticas públicas. 

La segunda lección trata de las reconfiguraciones del papel del Estado en el marco neoliberal, que promueven pérdidas en la gobernanza pública, financiación y regulación del sistema de salud ^46^ ^,^ ^47^ ^,^ ^48^. Una de las acciones más graves es debilitar y/o eliminar el rol regulador del Estado en salud desde la perspectiva neoliberal. En todos los sistemas de salud, incluso en los más liberales, el Estado, además de proveer servicios e insumos, regula a los agentes que integran el sistema de salud. Regular se encuentra directamente relacionado con la rectoría y visión estratégica del sector ^63^. 

La Organización Panamericana de la Salud (OPS) en el año 2020 publicó una nueva versión actualizada de las funciones esenciales de salud pública donde destaca la importancia del fortalecimiento de capacidades institucionales para la regulación ^64^. En el caso de Argentina, donde la fragmentación en el acceso deriva de la propia configuración institucional del sistema, es sumamente importante esta función. Si el Estado no regula, las consecuencias (obvias) son, por un lado, la escalada desmedida de precios de las cuotas de la medicina prepaga, los medicamentos y otros insumos. Por el otro lado, significa un quiebre en la lógica solidaria (fundamental para reducir inequidades) ^65^ del subsistema de la seguridad social que tradicionalmente ha cubierto a la mayor cantidad de población del país.

Desde la óptica neoliberal, se defiende la idea de que los usuarios del sistema puedan decidir dónde, cómo y con quién atenderse, pero se olvidan de mencionar que depende de la capacidad de pago de cada uno. Allí donde una decisión depende del dinero, deja de ser libre para estar fuertemente condicionada. Si esta lógica prevalece, la consecuencia será que quien no cuente con recursos dejará de buscar atención médica y, más allá de las terribles consecuencias individuales, esto impactará en la sociedad en su conjunto, aumentando la inequidad en el acceso, la ineficiencia y la injusticia del sistema.

## Consideraciones finales 

El sistema de salud argentino enfrenta múltiples desafíos inherentes a su diseño institucional y su carácter fragmentado y segmentado. La coexistencia de tres subsistemas con distintas lógicas de funcionamiento, cobertura y protección presenta obstáculos significativos para la articulación de estrategias sanitarias nacionales y la coordinación efectiva de las mismas. En la práctica, esto significa que los beneficiarios de cada subsistema tienen un acceso desigual a servicios en términos de tiempo y calidad. Además, el alto gasto en salud como porcentaje del PIB no se traduce en buenos resultados de salud, el elevado gasto de bolsillo es regresivo e impacta fuertemente en la economía de las familias más desfavorecidas, y hay una distribución desigual de recursos en centros asistenciales y profesionales.

Estos y otros problemas recurrentes del sistema han sido objeto de discusión a lo largo de la historia del país en el marco de debates en torno a la necesidad de reforma que, sin embargo, no han llegado a concretarse. Los factores que han influido en esta situación incluyen cuestiones políticas, el propio diseño institucional del sistema de salud, el papel central de los sindicatos en la seguridad social y su reticencia a introducir cambios que podrían socavar su posición en el sistema y las debilidades en la rectoría nacional frente a la marcada descentralización de la gestión. Como resultado, se han perpetrado varios subsistemas dentro del mismo país, lo que se traduce en fuertes desigualdades en la salud.

El análisis del caso argentino y su sistema de salud pone así de manifiesto la necesidad de una reorganización que priorice la universalización efectiva, garantizando la salud como un derecho y promoviendo el acceso equitativo a los servicios de salud en todo el país. Por el contrario, los cambios que podrían producirse desde una óptica ultraliberal asumen a la salud como una mercancía y transfieren la responsabilidad del Estado a los individuos. En lugar de fortalecer la oferta de servicios para garantizar una cobertura amplia y equitativa, estas propuestas se basan en una lógica de mercado donde la posibilidad de recibir atención médica depende de la capacidad de pago, lo que podría profundizar aún más las desigualdades existentes.

## Data Availability

Las fuentes de información utilizadas en el estudio se indican en el cuerpo del artículo.
